# Supplementary material for: African bushpigs exhibit porous species boundaries and appeared in Madagascar concurrently with human arrival
Source: Nat Commun. 2024 Jan 3;15:172. doi: 10.1038/s41467-023-44105-1 (PMC10764920; doi:10.1038/s41467-023-44105-1)
Supplement: Supplementary file 1 — Supplementary Information [file 41467_2023_44105_MOESM1_ESM.pdf]

African bushpigs exhibit porous species boundaries and appeared in Madagascar concurrently with human arrival

Authors

Renzo F. Balboa[1#], Laura D. Bertola[1#], Anna Brüniche-Olsen[1#], Malthe Sebro Rasmussen[1], Xiaodong Liu[1], Guillaume Besnard[2], Jordi Salmona[2], Cindy G. Santander[1], Shixu He[1], Dietmar Zinner[3,4,5], Miguel Pedrono[6], Vincent Muwanika[7], Charles Masembe[8], Mikkel Schubert[1,9], Josiah Kuja[1], Liam Quinn[1], Genís Garcia-Erill[1], Frederik Filip Stæger[1], Rianja Rakotoarivony[6], Margarida Henrique[10], Long Lin[1], Xi Wang[1], Michael P. Heaton[11], Timothy P. L. Smith[11], Kristian Hanghøj[1], Mikkel-Holger S. Sinding[1], Anagaw Atickem[12], Lounès Chikhi[2,10], Christian Roos[13], Philippe Gaubert[2,14], Hans R. Siegismund[1], Ida Moltke[1*], Anders Albrechtsen[1*] and Rasmus Heller[1*]

[1] Department of Biology, University of Copenhagen, Copenhagen, Denmark

[2] Laboratoire Evolution et Diversité Biologique (EDB), UMR 5174, CNRS, IRD, Université Toulouse Paul Sabatier, 31062 Toulouse, France

[3] Cognitive Ecology Laboratory, German Primate Center, Leibniz Institute for Primate Research, 37077 Göttingen, Germany

[4] Department of Primate Cognition, Georg-August-Universität Göttingen, 37077 Göttingen, Germany

[5] Leibniz Science Campus Primate Cognition, 37077 Göttingen, Germany

[6] UMR ASTRE, CIRAD, Campus International de Baillarguet, Montpellier, France

[7] College of Agricultural and Environmental Sciences, Makerere University, Kampala, Uganda

[8] College of Natural Sciences, Makerere University, Kampala, Uganda

[9] Novo Nordisk Foundation Center for Basic Metabolic Research, University of Copenhagen, Copenhagen, Denmark

[10] Instituto Gulbenkian de Ciência, Oeiras, Portugal

[11] USDA, ARS, US Meat Animal Research Center, Clay Center, Nebraska, USA

[12] Department of Zoological Sciences, Addis Ababa University, PO Box 1176, Addis Ababa, Ethiopia

[13] Gene Bank of Primates and Primate Genetics Laboratory, German Primate Center, Leibniz Institute for Primate Research, 37077 Göttingen, Germany

[14] Centro Interdisciplinar de Investigação Marinha e Ambiental (CIIMAR), Universidade do Porto, Terminal de Cruzeiros do Porto de Leixões, Av. General Norton de Matos, s/n, 4450-208, Porto, Portugal

[#] These authors contributed equally

* These authors jointly supervised this work

Supplementary Information

Supplementary Information **2**
Supplementary Figure 1. Estimated per-base sequencing error rates based on the 'perfect individual' approach for samples mapped to the common warthog reference genome 3
Supplementary Figure 2. KING kinship coefficients for two Ethiopian and two Equatorial Guinean samples that were merged based on 2D-SFS 3
Supplementary Figure 3. Inferred ancestry proportions for unrelated samples, excluding Madagascar (n = 33) using NGSadmix, assuming 2 (K = 2) ancestral populations 3
Supplementary Figure 4. Inferred ancestry proportions for 54 unrelated samples using NGSadmix, assuming 2 (K = 2) to 8 (K = 8) ancestral populations 5
Supplementary Figure 5. Hudson's F_{ST} for 18 medium-high depth individuals 6
Supplementary Figure 6. f-branch (f_b) statistics for all 13 populations 6
Supplementary Figure 7. Estimation of admixture proportions (f_4-ratios) between populations 7
Supplementary Figure 8. Runs of homozygosity estimation using ROHan for all individuals (n = 67) 8
Supplementary Figure 9. Mitochondrial phylogeny based on complete mitochondrial genomes using BEAST 8

Supplementary Figures

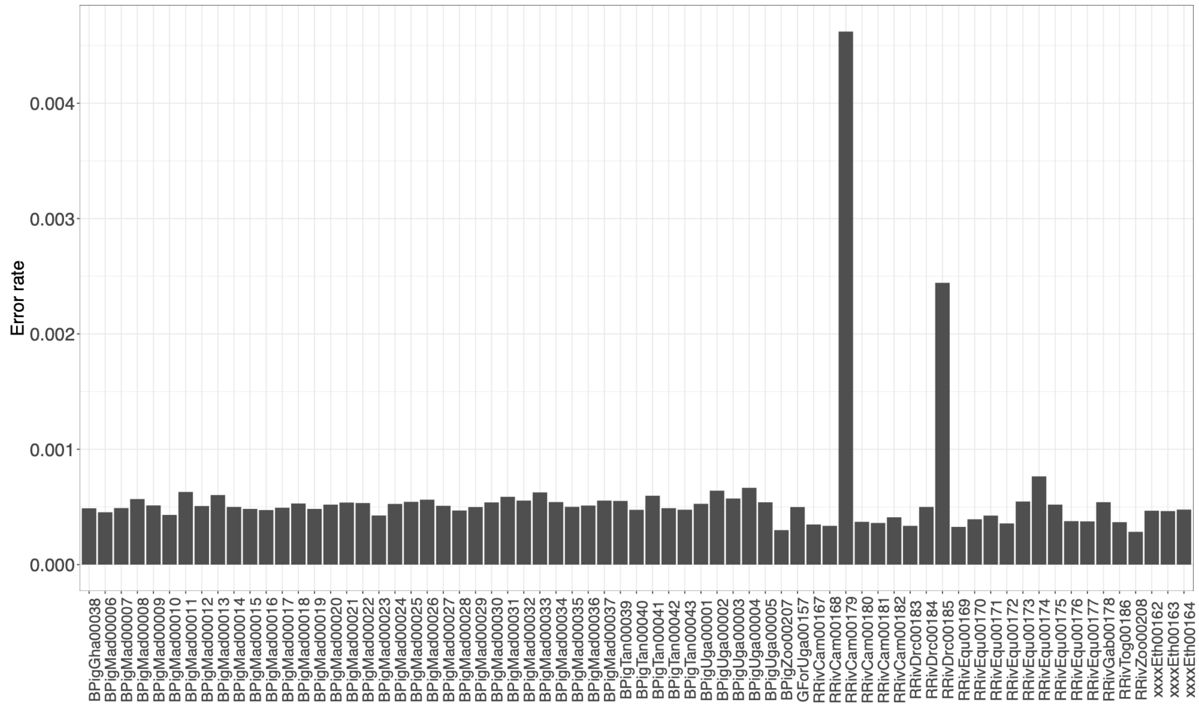

Supplementary Figure 1. Estimated per-base sequencing error rates based on the 'perfect individual' approach for samples mapped to the common warthog reference genome.

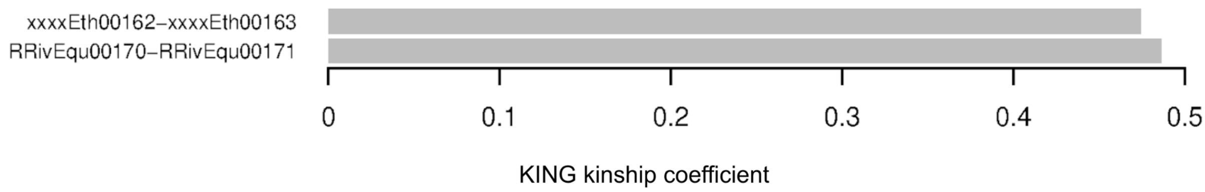

Supplementary Figure 2. KING kinship coefficients for two Ethiopian and two Equatorial Guinean samples that were merged based on 2D-SFS.

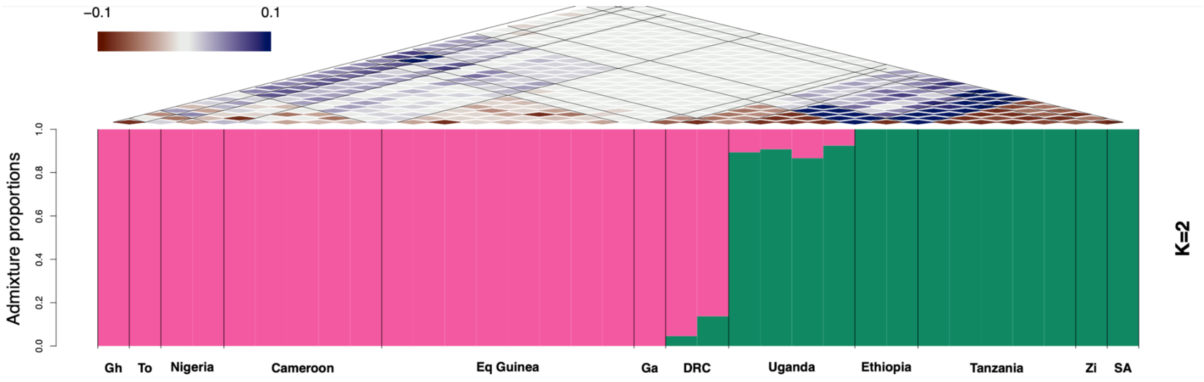

Supplementary Figure 3. Inferred ancestry proportions for unrelated samples, excluding Madagascar (n = 33) using NGSadmix, assuming 2 (*K* = 2) ancestral populations. The barplot indicates admixture proportions for 2 ancestral populations, while triangles above indicate pairwise correlations of residuals as assessed by evalAdmix. Gh – Ghana, To – Togo, Ga – Gabon, DRC – Democratic Republic of Congo, Zi – Zimbabwe, SA – South Africa.

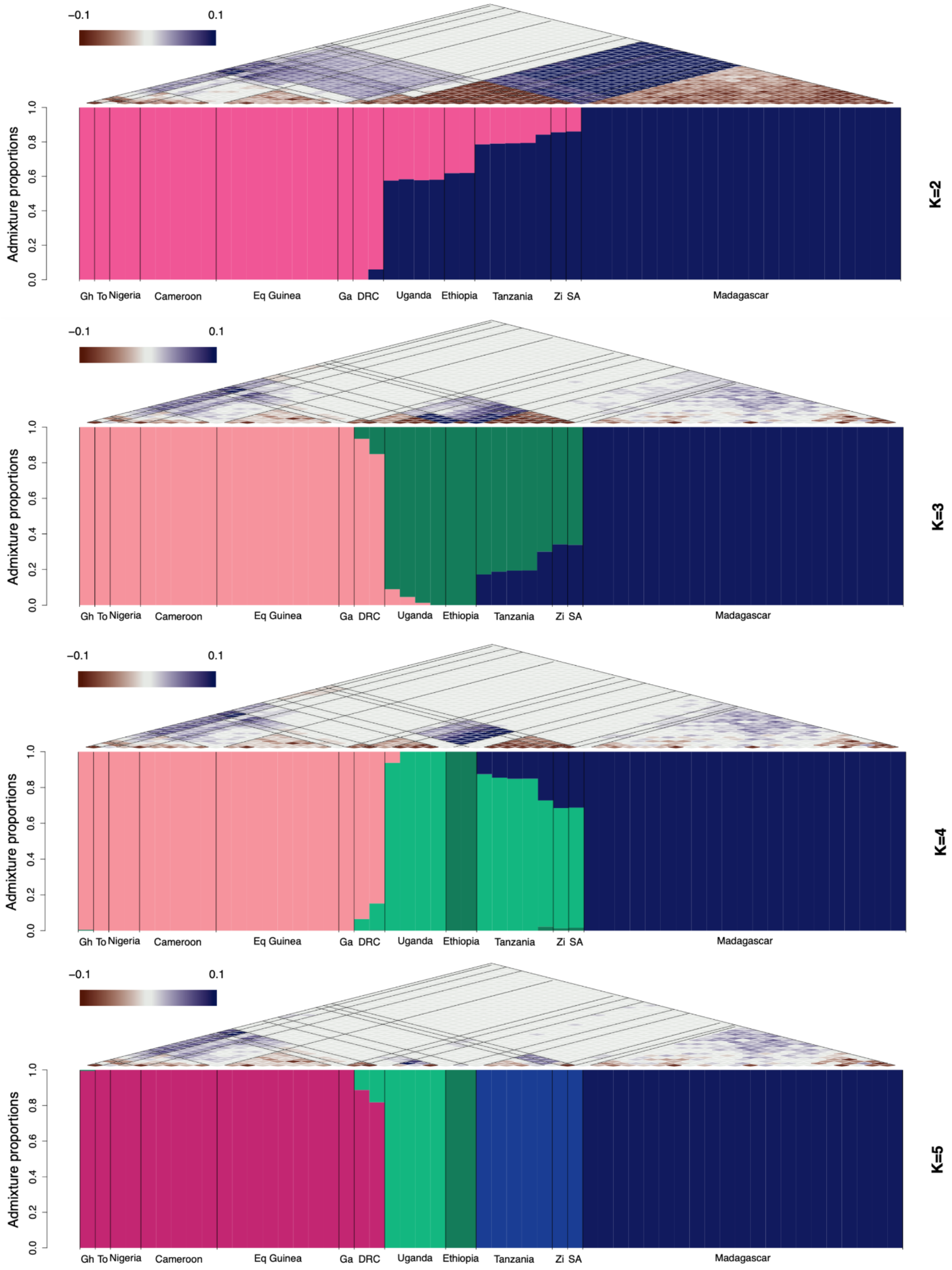

−0.1
0.1
Admixture proportions
0.0 0.2 0.4 0.6 0.8 1.0
Gh To Nigeria Cameroon Eq Guinea Ga DRC Uganda Ethiopia Tanzania Zi SA Madagascar
K=2
K=3
K=4
K=5

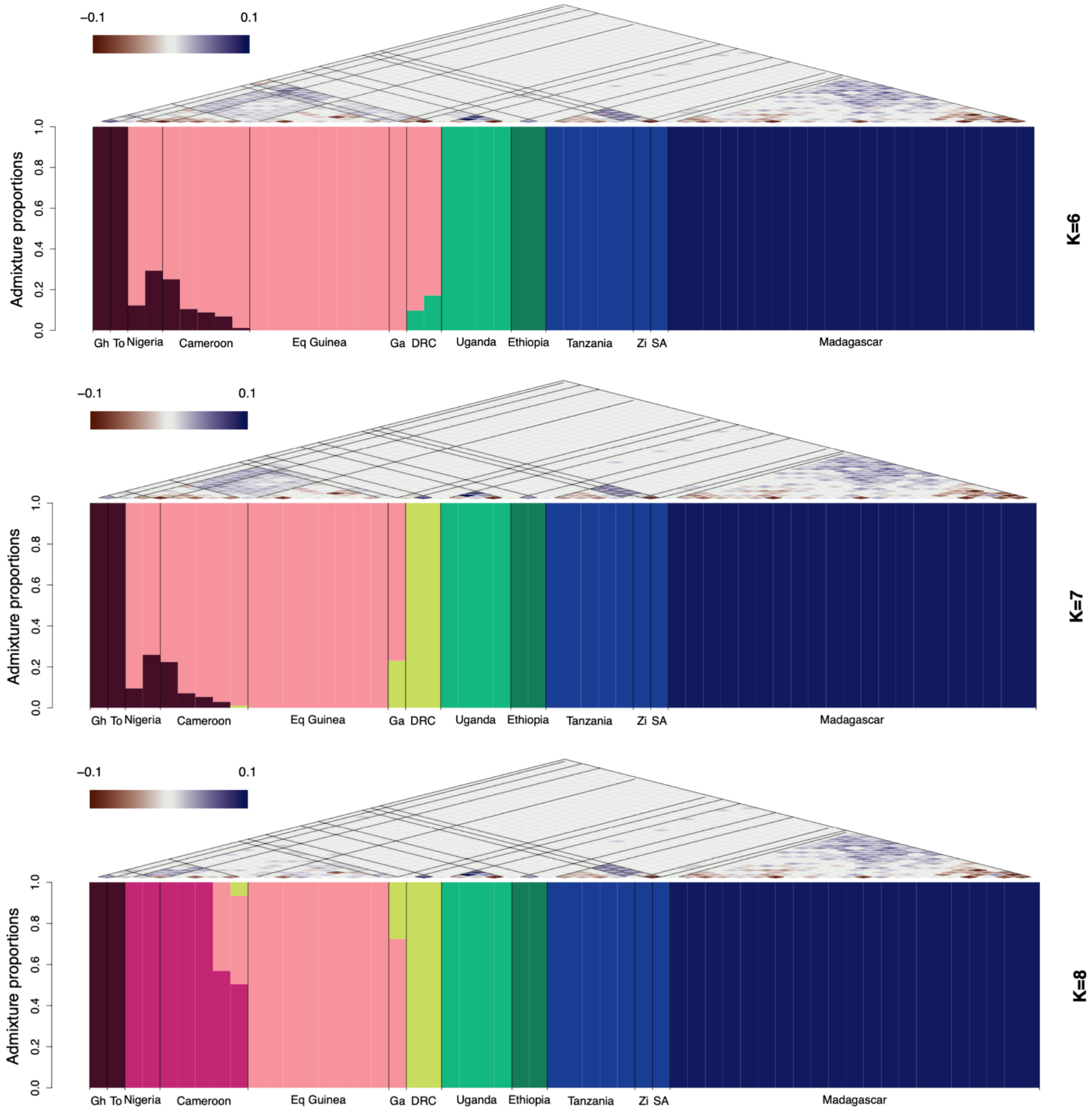

Supplementary Figure 4. Inferred ancestry proportions for 54 unrelated samples using NGSadmix, assuming 2 (*K* = 2) to 8 (*K* = 8) ancestral populations. Barplots indicate admixture proportions for each number of assumed ancestral populations, while triangles above each barplot indicate pairwise correlations of residuals as assessed by evalAdmix. Gh – Ghana, To – Togo, Ga – Gabon, DRC – Democratic Republic of Congo, Zi – Zimbabwe, SA – South Africa.

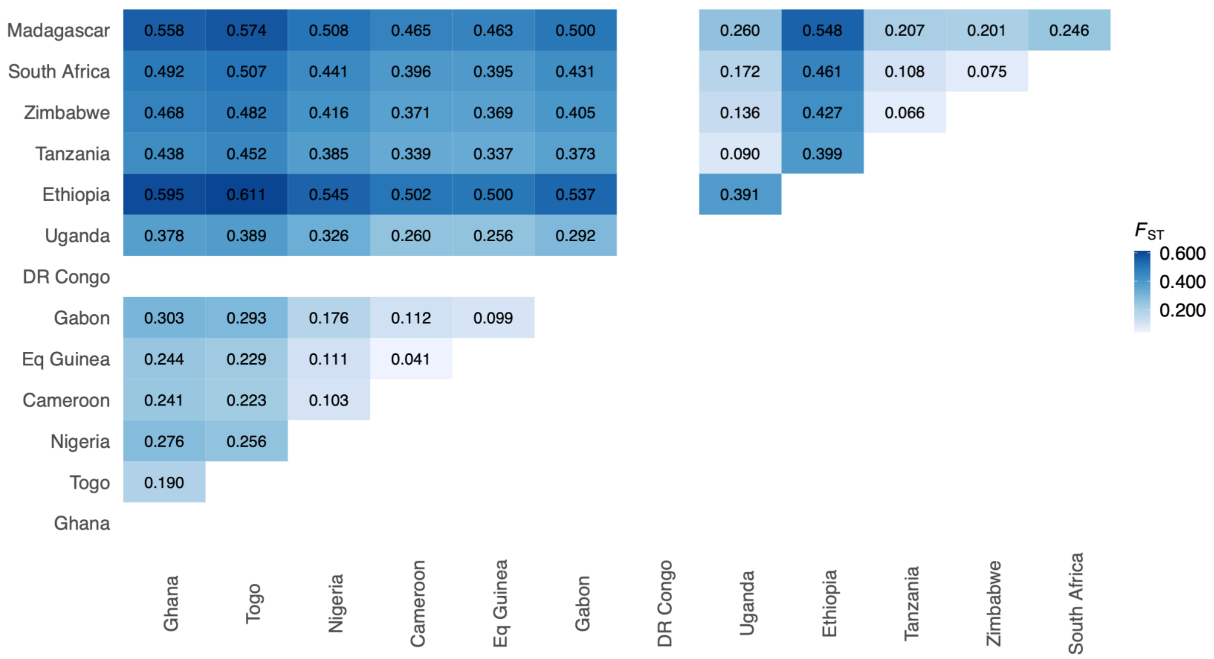

Supplementary Figure 5. Hudson's F_{ST} for 18 medium-high depth individuals.

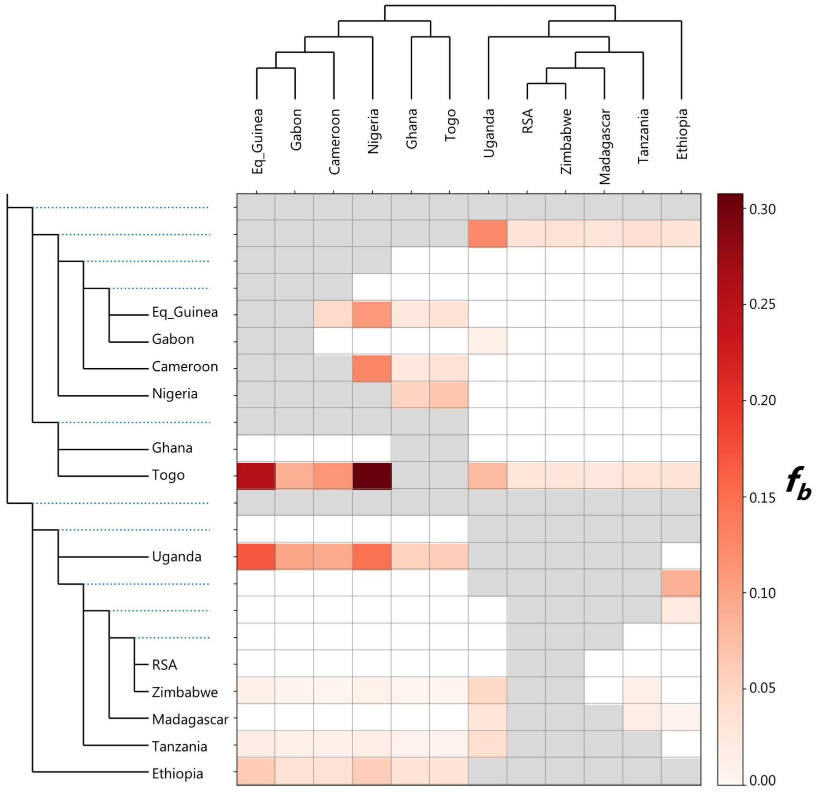

Supplementary Figure 6. *f*-branch (f_b) statistics for all 13 populations. Heatmap describing the extent of allele sharing between the corresponding branch of the species tree on the y axis, relative to its sister branch, and populations on the x-axis. RSA – South Africa.

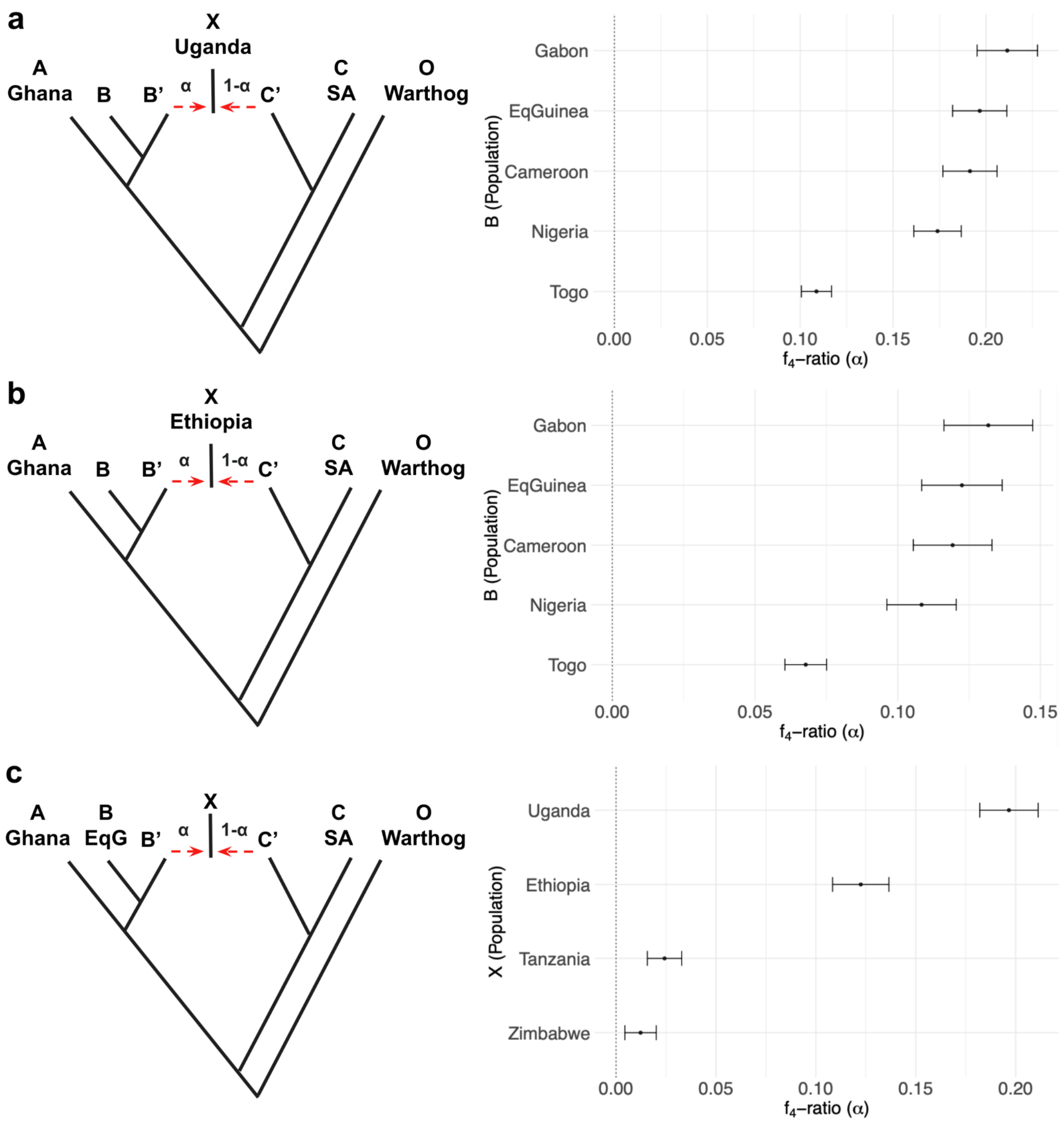

Supplementary Figure 7. Estimation of admixture proportions (f_4-ratios) between populations. f_4-ratios using medium-high depth individuals (≥14×; n = 18) were calculated using the common warthog as an outgroup, and constructed as f_4(A,O;X,C)/f_4(A,O;B,C). a) f_4-ratios into Uganda from non-Ghanian red river hog populations (B), of the form (f_4(Ghana, Warthog; Uganda, South Africa)/f_4(Ghana, Warthog; B, South Africa). b) f_4-ratios into Ethiopia from non-Ghanian red river hog populations (B) (f_4(Ghana, Warthog; Ethiopia, South Africa)/f_4(Ghana, Warthog; B, South Africa). c) f_4-ratios into bushpig populations (X), using Equatorial Guinea as a proxy. α indicates estimated admixture proportions from B' into X (f_4(Ghana, Warthog; X, South Africa)/f_4(Ghana, Warthog; Eq Guinea, South Africa)). Data are presented as f_4-ratios ± three standard errors. SA – South Africa; EqG – Equatorial Guinea.

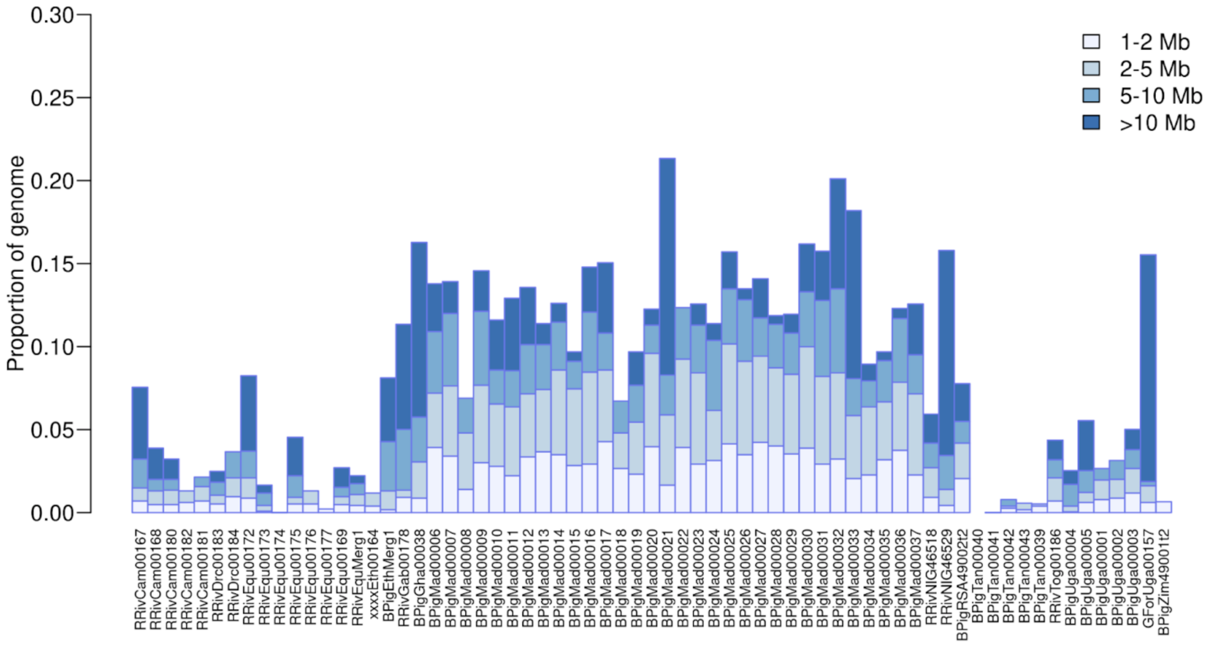

Supplementary Figure 8. Runs of homozygosity estimation using ROHan for all individuals (n = 67).

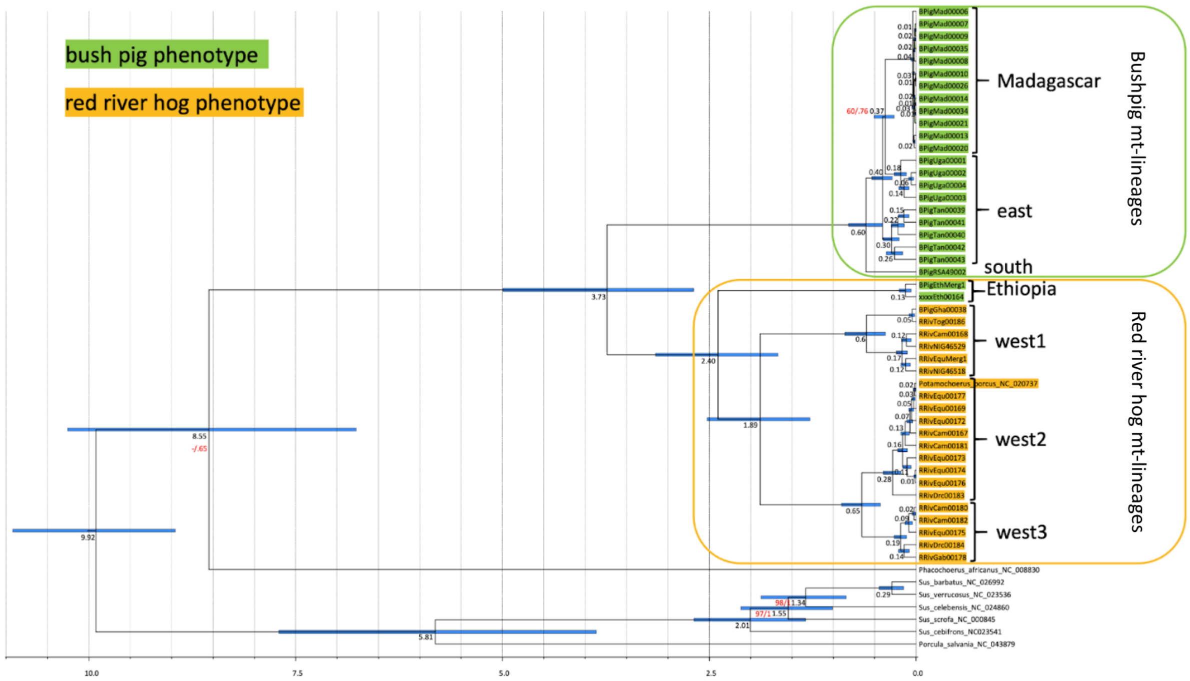

Supplementary Figure 9. Mitochondrial phylogeny based on complete mitochondrial genomes using BEAST.
