# Supplementary material for: African bushpigs exhibit porous species boundaries and appeared in Madagascar concurrently with human arrival
Source: Nat Commun. 2024 Jan 3;15:172. doi: 10.1038/s41467-023-44105-1 (PMC10764920; doi:10.1038/s41467-023-44105-1)
Supplement: Supplementary file 3 — Description of Additional Supplementary Files [file 41467_2023_44105_MOESM3_ESM.pdf]

Description of Additional Supplementary Files

Supplementary Data

Supplementary Data 1. Overview of samples used in this study. Among 71 (+2 merged) pig samples used within this study, 67 passed quality filters and were included in population genetic analyses (see 'Include – all individuals'). Two other filters, relatedness ('Include – unrelated'; n = 54) and medium-high depth (≥14X; 'Include – medium-high depth and unrelated'; n = 18) were also used. Relationships were determined by KING and R0/R1 analyses (see 'Exclusion criteria/Notes' for more information). Two previously unpublished outgroups used for polarisation are also included here. FS – Full-siblings, PO – Parent-offspring.

Supplementary Data 2. Summary of site filters for the common warthog genome.

Supplementary Data 3. Summary of datasets and samples used for analyses in this study.

Supplementary Data 4. Identical haplotypes removed in mtDNA phylogenetic reconstructions.
