# Supplementary material for: African bushpigs exhibit porous species boundaries and appeared in Madagascar concurrently with human arrival
Source: Nat Commun. 2024 Jan 3;15:172. doi: 10.1038/s41467-023-44105-1 (PMC10764920; doi:10.1038/s41467-023-44105-1)
Supplement: Supplementary file 8 — Reporting Summary [file 41467_2023_44105_MOESM8_ESM.pdf]

nature portfolio

Corresponding author(s): Heller

Last updated by author(s): Nov 20, 2023

Reporting Summary

Nature Portfolio wishes to improve the reproducibility of the work that we publish. This form provides structure for consistency and transparency in reporting. For further information on Nature Portfolio policies, see our Editorial Policies and the Editorial Policy Checklist.

Statistics

For all statistical analyses, confirm that the following items are present in the figure legend, table legend, main text, or Methods section.

n/a	Confirmed	
☐	☒	The exact sample size (*n*) for each experimental group/condition, given as a discrete number and unit of measurement
☐	☒	A statement on whether measurements were taken from distinct samples or whether the same sample was measured repeatedly
☒	☐	The statistical test(s) used AND whether they are one- or two-sided<br>*Only common tests should be described solely by name; describe more complex techniques in the Methods section.*
☒	☐	A description of all covariates tested
☒	☐	A description of any assumptions or corrections, such as tests of normality and adjustment for multiple comparisons
☐	☒	A full description of the statistical parameters including central tendency (e.g. means) or other basic estimates (e.g. regression coefficient) AND variation (e.g. standard deviation) or associated estimates of uncertainty (e.g. confidence intervals)
☐	☒	For null hypothesis testing, the test statistic (e.g. *F*, *t*, *r*) with confidence intervals, effect sizes, degrees of freedom and *P* value noted<br>*Give P values as exact values whenever suitable.*
☒	☐	For Bayesian analysis, information on the choice of priors and Markov chain Monte Carlo settings
☐	☒	For hierarchical and complex designs, identification of the appropriate level for tests and full reporting of outcomes
☒	☐	Estimates of effect sizes (e.g. Cohen's *d*, Pearson's *r*), indicating how they were calculated

Our web collection on statistics for biologists contains articles on many of the points above.

Software and code

Policy information about availability of computer code

Data collection	Data generation and collection is described in Methods. A description of data used within this study is provided within Supplementary Data 1. Note that the tissue and DNA samples are sourced from pre-existing scientific collections. Therefore, no field collection was carried out specifically for the present study, although Microsoft Excel and basic bash scripts were used to collate metadata for samples utilised within this study.
Data analysis	Code used for analyses are publicly available on Github: https://github.com/popgenDK/seqAfrica_bushpigs. Software used and their version numbers are described in Methods and provided here: FastQC v0.11.9; MultiQC v1.13; PALEOMIX BAM pipeline (branch 'pub/2022/africa'); AdapterRemoval v2.3.2; BWA-mem v0.7.17-r1188; samtools v1.11; bcftools v1.13; GENMAP v1.3.0; RepeatMasker v4.1.1; Angsd v0.925; PCAngsd v1.02; NGSadmix (2020.04.20); winsfs v0.7.0; EEMS v0.0.0.9000; Dsuite v0.5-r44; ADMIXTOOLS v7.0.2; PSMC v0.6.5; PLINK v1.9; ROHan v1.0.1; popSizeABC (2016.10.20); Geneious v2023.0.1; BBDuk v37.64; Dedupe v37.64; Muscle v3.8.31; AliView v1.18; IQ-TREE v2.2.0; BEAST v2.6.7; Tracer v1.6; FigTree v1.4.2.

For manuscripts utilizing custom algorithms or software that are central to the research but not yet described in published literature, software must be made available to editors and reviewers. We strongly encourage code deposition in a community repository (e.g. GitHub). See the Nature Portfolio guidelines for submitting code & software for further information.

Data

Policy information about availability of data

All manuscripts must include a data availability statement. This statement should provide the following information, where applicable:

- Accession codes, unique identifiers, or web links for publicly available datasets
- A description of any restrictions on data availability
- For clinical datasets or third party data, please ensure that the statement adheres to our policy

Data used in this study are described within the Article, Supplementary Data and Supplementary Information. Sample locations and their sources are described within Supplementary Data 1. Raw fastq files generated inused within this study and their associated metadata are publicly available on the NCBI database under BioProject accession PRJNA1027560 [https://www.ncbi.nlm.nih.gov/bioproject/PRJNA1027560/]. BioSample accessions for each sample are described within Supplementary Data 1. Raw data from Xie et al., available on the NGDC database (SAMC146518 [https://ngdc.cncb.ac.cn/biosample/browse/SAMC146518] and SAMC146529 [https://ngdc.cncb.ac.cn/biosample/browse/SAMC146529]), a chromosome-level assembly of Phacochoerus africanus, available on the NCBI database (GCA_016906955.1 [https://www.ncbi.nlm.nih.gov/datasets/genome/GCF_016906955.1/]) and reads from Sus scrofa (SAMN28197093 [https://www.ncbi.nlm.nih.gov/biosample/SAMN28197093]) were also used in this study. Additional mitogenome sequences available in GenBank were utilised for mitochondrial analyses (P. porcus: NC_020737 [https://www.ncbi.nlm.nih.gov/nuccore/NC_020737], Ph. africanus: NC_008830 [https://www.ncbi.nlm.nih.gov/nuccore/NC_008830], Porcula salvania: NC_043879 [https://www.ncbi.nlm.nih.gov/nuccore/NC_043879], Sus scrofa: NC_000845 [https://www.ncbi.nlm.nih.gov/nuccore/NC_000845], S. cebifrons: NC_023541 [https://www.ncbi.nlm.nih.gov/nuccore/NC_023541], S. celebensis: NC_024860 [https://www.ncbi.nlm.nih.gov/nuccore/NC_024860], S. barbatus: NC_026992 [https://www.ncbi.nlm.nih.gov/nuccore/NC_026992], S. verrucosus: NC_023536 [https://www.ncbi.nlm.nih.gov/nuccore/NC_023536]). Source data are provided as a Source Data file.

Research involving human participants, their data, or biological material

Policy information about studies with human participants or human data. See also policy information about sex, gender (identity/presentation), and sexual orientation and race, ethnicity and racism.

Reporting on sex and gender	n.a.
Reporting on race, ethnicity, or other socially relevant groupings	n.a.
Population characteristics	n.a.
Recruitment	n.a.
Ethics oversight	n.a.

Note that full information on the approval of the study protocol must also be provided in the manuscript.

Field-specific reporting

Please select the one below that is the best fit for your research. If you are not sure, read the appropriate sections before making your selection.

☐ Life sciences ☐ Behavioural & social sciences ☒ Ecological, evolutionary & environmental sciences

For a reference copy of the document with all sections, see nature.com/documents/nr-reporting-summary-flat.pdf

Ecological, evolutionary & environmental sciences study design

All studies must disclose on these points even when the disclosure is negative.

Study description	Investigation of population histories of African bushpigs and red river hogs. Here, we investigate 67 samples from 13 populations, including red river hogs (Ghana (n=1); Togo (n=1); Nigeria (n=2); Cameroon (n=5); Equatorial Guinea (n=8); Gabon (n=1); DR Congo (n=2)) and African bushpigs (Ethiopia (n=2); Uganda (n=6); Tanzania (n=5); Zimbabwe (n=1); South Africa (n=1) and Madagascar (n=32)). Depending on analyses, samples were treated as individual-level units (n=18, n=54, or n=67 depending on the analysis performed) or population-level units (n=13).
Research sample	Samples are from the genus Potamochoerus, chosen because the study aim was to investigate population histories of bushpigs and red river hogs, and are therefore the taxa of interest. Samples were sourced from 13 different locations, each representing a separate population - from Ghana, Togo, Nigeria, Cameroon, Equatorial Guinea, Gabon, DR Congo, Ethiopia, Uganda, Tanzania, Zimbabwe, South Africa and Madagascar. Exact location coordinates can be found in Supplementary Data 1 and in Source Data. Samples were then sequenced (dedscribed in Methods), generating DNA sequencing data which were the raw datasets that were subsequently analysed. In addition, publicly available data was used - raw data from Xie et al. (NCBI BioSample accessions: SAMC146518 and SAMC146529) and a chromosome-level assembly of Phacochoerus africanus (accession: GCA_016906955.1) were also used in this study, described in Supplementary Data 1. Additional mitogenome sequences available in GenBank were also utilised for mitochondrial analyses (Potamochoerus porcus: NC_020737, Phacochoerus africanus: NC:008830, Porcula salvania: NC_043879,

	Sus scrofa: NC_000845, S. cebifrons: NC_023541, S. celebensis: NC_026992, S. barbatus: NC_026992, S. verrucosus: NC_023536). Source data used to generate figures are provided as a Source Data file.
Sampling strategy	No sample size calculations were performed, but were chosen based on availability and breadth across different countries in Africa to answer research questions described in the Introduction. Tissue and DNA samples were sourced from pre-existing scientific collections, and were contributed by different co-authors. The dataset was assembled opportunistically without any coherent sample collection strategy, due to the sparse availability of relevant material for the investigated taxon. Therefore, no field collection was carried out specifically for the present study.
Data collection	Samples were gathered from scientific collections at different institutions (described in Supplementary Table 1) and contributed by different co-authors. Sample contributions and metadata were recorded by members in labs within and/or were collaborators with researchers from CIRAD, Makere University, EDB, Addis Ababa University, the USDA and the University of Copenhagen (detailed further in Supplementary Data 1 and the Author Contributions Statement). In particular, Komlan Afiademanyo (Université de Lomé, Togo), Flobert Njikou (Université de Yaoundé I, Cameroon), Alain Didier Missoup (Université de Douala), Gabriel Ngua (ANDEGE, Equatorial Guinea), and Jonas Muhindo and Idriss Ayaya (CIFOR, Democratic Republic of Congo) collected samples in Western and Central Africa. Peter Arctander organised sample collections between the 1980s and 1990s and David Moyer who contributed samples collected between 1995-1997 and subsequently stored in the collection at the University of Copenhagen. Existing information was documented through a combination of mechanical means and data entry into relevant sheets. We are also grateful to collaborators from African wildlife management authorities granted express permission to use samples within this study.
Timing and spatial scale	The dataset was assembled opportunistically without any coherent sample collection strategy, due to the sparse availability of relevant material for the investigated taxon. Data was primarily collected from the 1990s onwards, particularly between 1995-1997 and were collected from 13 different locations - Ghana, Togo, Nigeria, Cameroon, Equatorial Guinea, Gabon, DR Congo, Ethiopia, Uganda, Tanzania, Zimbabwe, South Africa and Madagascar. Exact location coordinates are detailed in Supplementary Data 1.
Data exclusions	As described in Results, Two red river hog samples, from Cameroon and DR Congo, were excluded due to high sequencing error rates (Supplementary Fig. S1).
Reproducibility	Most populations contain multiple samples that were analysed, and some analyses have been replicated. Fst was independently calculated using two different sample sets - one for all individuals (Fig. 2a) and high-depth individuals only (Supp. Fig. S5). NGSadmix with K = 2 to K = 9 was independently replicated up to 4000 times until the model converged, defined as when the top 3 maximum likelihood runs were within 10 log-likelihood units of each other or until a limit of 4000 independent runs was reached without convergence. EEMS was run using 300 demes for three independent runs of 30 million iterations, discarding the first 15 million as burn-in. For popSizeABC analyses, 210,000 simulations were performed for 100 2Mb regions per simulation as per the suggested settings in the popSizeABC publication for the software. For other analyses, results were primarily dependent on the underlying data. Assuming that the data are treated and analysed appropriately, results for these analyses are reproducible.
Randomization	Samples were initially grouped based on location, and were then classified into populations after investigating population structure between samples.
Blinding	Blinding was not used within this study, but analyses for all samples were treated the same after sample exclusion, described in Methods. Blinding was not performed because this study does not measure or evaluate a exposure.

Did the study involve field work? ☐ Yes ☒ No

Reporting for specific materials, systems and methods

We require information from authors about some types of materials, experimental systems and methods used in many studies. Here, indicate whether each material, system or method listed is relevant to your study. If you are not sure if a list item applies to your research, read the appropriate section before selecting a response.

Materials & experimental systems

n/a	Involved in the study	
☒	☐	Antibodies
☒	☐	Eukaryotic cell lines
☒	☐	Palaeontology and archaeology
☐	☒	Animals and other organisms
☒	☐	Clinical data
☒	☐	Dual use research of concern
☒	☐	Plants

Methods

n/a	Involved in the study	
☒	☐	ChIP-seq
☒	☐	Flow cytometry
☒	☐	MRI-based neuroimaging

Animals and other research organisms

Policy information about studies involving animals; ARRIVE guidelines recommended for reporting animal research, and Sex and Gender in Research

Laboratory animals	n.a.

Wild animals	The tissue and DNA samples are sourced from-pre-existing scientific collections, and therefore no field collection was carried out specifically for the present study.
Reporting on sex	n.a.
Field-collected samples	Details about sample storage are provided in the Methods section in the manuscript.
Ethics oversight	All samples were provided directly by scientists (co-authors) from pre-existing scientific collections. Samples were collected complying with local and international legislation. All sample collection predates the Nagoya protocol. The research was carried out in compliance with the Code of Conduct for Responsible Research of the University of Copenhagen.

Note that full information on the approval of the study protocol must also be provided in the manuscript.
